# Editorial: Methods, tools and algorithms in data visualization bioinformatics

**DOI:** 10.3389/fbinf.2026.1975924

**Published:** 2026-09-22

**Authors:** Erqiang Hu

**Affiliations:** Department of Pathology, Albert Einstein College of Medicine, Bronx, NY, United States

**Keywords:** algorithms, bioinformatics, computational tools, data visualization, visual analytics

Large and structurally complex biological datasets cannot be interpreted from numerical summaries alone. Visualization is part of the analysis itself: researchers use it to assess data quality, detect patterns, interpret results, and communicate findings. The seven articles in this Research Topic examine these functions in genomics, epigenomics, transcriptomics, peptidomics, pathogen surveillance, molecular design, and mesoscale cell biology. Although their applications differ, each uses a domain-specific visual interface to bring computational output into a form that supports biological judgment.

Three articles present integrated workflows for high-dimensional omics data. GeneCloudOmics combines 23 analysis and visualization tasks in a web interface, including normalization, exploratory plots, dimensionality reduction, clustering, differential expression, enrichment, and protein-level analyses (Helmy et al.). Keeping preprocessing, quality assessment, statistical analysis, and visualization in the same environment may make the workflow more accessible to researchers who do not routinely use command-line tools. Peptimetric addresses a similar problem in mass-spectrometry-based peptidomics. Users can compare proteins and peptides interactively across samples, moving from sample-level summaries to patterns for individual peptides (Hartman et al.). ChromTag applies this integrated approach to CUT&Tag and ChIP-seq peak count data. It combines differential peak analysis, genomic annotation, functional and motif enrichment, and views that include volcano plots, principal component analysis, heatmaps, and genomic profiles (Xu et al.). In all three tools, the visual outputs remain linked to the analytical steps and choices that produced them.

Two articles use visualization to guide operational or experimental decisions. RIPS converts NCBI Pathogen Detection Rapid Reports and associated metadata into an interactive dashboard for monitoring foodborne bacterial pathogens (Muruvanda et al.). Users can apply consistent, adjustable criteria and then inspect the isolates behind a potential signal. This design organizes genomic distance and contextual metadata around the surveillance question, rather than simply rendering text reports as plots. ApE supports a different kind of decision: planning and checking molecular biology experiments (Davis and Jorgensen). Annotated sequence views and linear or circular plasmid maps are linked to simulations of PCR, restriction-ligation, Gibson, and Golden Gate assembly, together with tools for primer design and sequence validation. The visualization therefore becomes part of the experimental workflow, not merely its documentation.

Two other contributions extend visualization beyond primary data analysis. pubCounteR connects experimentally derived gene lists with the literature by querying PubMed using biological terms chosen by the user and summarizing publication activity for each gene (Leer et al.). Researchers can distinguish genes with an extensive literature from candidates that have received little attention in the chosen context, while retaining a direct route to the underlying publications. CellPAINT works at a different scale (Gardner et al.). It allows non-expert users to build molecular illustrations of cells and viruses and to add custom components derived from Protein Data Bank structures. In its application to cryoelectron tomography, these illustrations can help users organize existing knowledge, make spatial assumptions explicit, and develop hypotheses about cellular environments that are only partly observed.

Despite differences in data type and purpose, the seven articles converge on several practical requirements. A useful visual system should reflect the structure of its biological subject, let users move between overview and detail, connect displays to filtering, annotation, or statistical analysis, and support inspection and export of results. Most tools also aim to improve accessibility. A graphical interface can lower technical barriers, but it should not hide decisions about normalization, thresholds, identifiers, reference databases, or uncertainty. Those choices need to remain visible so that users can relate an interpretation to the data and analysis that produced it.

The articles also point to several areas that deserve more attention: systematic testing of usability and interpretive accuracy, clearer treatment of uncertainty and missing data, and better support for provenance, versioning, and reproducible export. As datasets and workflows grow more heterogeneous, visual tools will also need to work more readily with established analysis pipelines. Evaluation should focus on whether a system helps users reach defensible conclusions and explain how they were obtained; simply counting plots or algorithms says little about that goal.

We thank the authors, reviewers, and editorial staff for their work on this Research Topic. We hope that researchers will find the Research Topic useful when selecting tools for current studies, and that developers will draw on it when designing transparent, task-centered visual analytics for bioinformatics.

